# Decreased cytotoxic T cells and TCR clonality in organ transplant recipients with squamous cell carcinoma

**DOI:** 10.1038/s41698-020-0119-9

**Published:** 2020-06-03

**Authors:** Nicholas Frazzette, Alireza Khodadadi-Jamayran, Nicole Doudican, Alexis Santana, Diane Felsen, Anna C. Pavlick, Aristotelis Tsirigos, John A. Carucci

**Affiliations:** 1 Ronald O. Perelman Department of Dermatology, New York University Langone Medical Center, New York, NY USA; 2 Applied Bioinformatics, New York University Langone Medical Center, New York, NY USA; 3 Department of Pediatric Urology, Weill Medical College of Cornell, New York, USA; 4 Perlmutter Cancer Center, NYU Langone Medical Center, New York, NY USA

**Keywords:** Squamous cell carcinoma, Cancer microenvironment, Squamous cell carcinoma, Tumour immunology

## Abstract

T-cell landscape differences between cutaneous squamous cell carcinoma (cSCC) tumors in immune competent (SCC in IC) and immunocompromised organ transplant recipients (TSCC in OTR) are unclear. We developed an analytical method to define tumor infiltrating lymphocyte (TIL) phenotype in cSCC from immune competent and immune suppressed patients using single-cell TCR sequencing and gene expression data. TSCC exhibits reduced proportions of cytotoxic and naïve TILs and similar numbers of regulatory TILs. Fewer, more heterogeneous TCR clonotypes are observed in TIL from OTR. Most TCR sequences for top ten clonotypes correspond to known antigens, while 24% correspond to putative neoantigens. OTR show increased cSCC events over 12 months possibly due to reduced cytotoxic T-cells. Our novel method of barcoding CD8+ T-cells is the first providing gene expression and TCR sequences in cSCC. Knowledge regarding putative antigens recognized by TCRs with phenotypic function of T-cells bearing those TCRs could facilitate personalized cSCC treatments.

## Introduction

Cutaneous squamous cell carcinoma (cSCC) is the second most common human skin cancer, accounting for 20–50% of all skin cancer diagnoses^1^. Although usually curable surgically, cSCC may behave aggressively with 2–5% of tumors eventuating in nodal metastases. Immunosuppression is a key risk factor for the development of cSCC development and poor outcomes, including nodal metastasis and disease-specific death^2^. The importance of the tumor immune microenvironment, and particularly the T cell microenvironment, in cSCC is underscored by the fact that PD-1 checkpoint inhibition results in tumor regression in immunocompetent patients, even those with metastatic disease^3–5^. The immune microenvironment in transplant-associated SCC (TSCC) differs from cSCC in immune competent patients. TSCC shows a higher regulatory T cell (Treg)/cytotoxic T cell (Tc) ratio believed to favor immune evasion by TSCC^6^. Clearly, T cell phenotype and function are important in determining host response and ultimately outcomes from cSCC^7^.

## Results and discussion

### Single-cell RNAseq and TCR sequencing are used together to define the T-cell landscape in cSCC

To gain further insight into the immune microenvironment in cSCC, we obtained CD8+T cells from fresh cSCC samples obtained from immunocompetent and immunocompromised transplant patients. These T cells were subject to single-cell RNA seq to characterize distinct T cell populations based on gene expression profiles. Additionally, the α and β CDR3 regions of the TCR was sequenced to characterize the T cell immune response in these patients. Barcoding was used to correlate gene expression to TCR sequence on a single cell basis. We analyzed data using iCellR, a custom R package we developed for normalizing, clustering, and visualizing single cell RNA sequencing data and VDJ data of TCRs from single cell sequencing^8^. It builds on previous programs designed for RNA sequencing analysis and VDJ sequencing analysis by handling a barcoding function which allows individual VDJ sequences to be matched with individual gene expression signatures.

### Patient demographics

Five SCC samples and six TSCC samples were analyzed in this study. All tumors samples were American Joint Committee on Cancer (AJCC) stage 2 and Brigham and Women’s hospital (BWH) stage 2A. The male:female ratio was 3:2 in SCC and 1:5 in TSCC. Mean patient age was 81 years in SCC and 65 years in TSCC. TSCC2 and TSCC3 were obtained from the same patient (Supplementary Table 1). OTRs in our study showed increased numbers of cSCC events over 12 months (7.2 vs. 1.2 patients, *p* < 0.01, Supplementary Table 1). OTRs underwent either liver or kidney transplant and all were on 2 or 3 drug immune suppression protocols, including combinations of mycophenolate, tacrolimus, sirolimus, cyclosporine, azathioprine and/or prednisone (Supplementary Table 1). Average length of immune suppression is 17.6 years. Three of five transplant recipients (TSCC1, 2/3, 4) met criteria for catastrophic carcinomatosis^9^. Average length of immune suppression for catastrophic patients was 24.6 years vs. 9 years (Supplementary Table 1).

### cSCC tumors from solid organ transplant patients exhibit reduced clonality

**Fig. 1:**
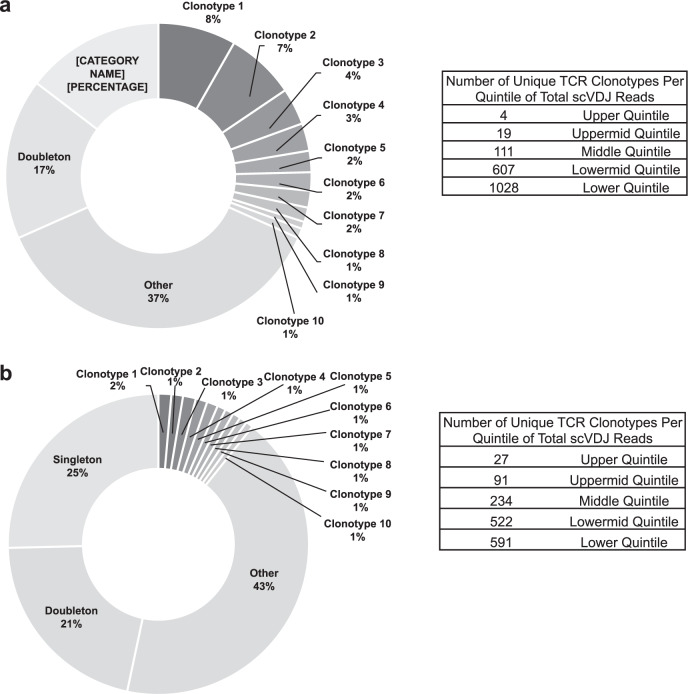
TCR clonality for TIL from human squamous cell carcinoma. **a** Percent of total scVDJ sequencing reads represented by unique clonotypes. Clonotypes 1–10 are the most frequently appearing clonotypes in SCC3. Doubletons are all clonotypes appearing twice and singletons are all clonotypes appearing once in SCC3. Other is all tripletons or higher, excluding the top ten most frequent clonotypes. **b** Percent of total scVDJ sequencing reads represented by unique clonotypes. Clonotypes 1–10 are the most frequently appearing clonotypes in TSCC5. Doubletons are all clonotypes appearing twice and singletons are all clonotypes appearing once in TSCC5. Other is all tripletons or higher, excluding the top ten most frequent clonotypes.

The total number of clonotypes per tumor and total number of cells analyzed per tumor were analyzed. TILs from both SCC and TSCC exhibited clonality with many highly expanded single receptor clone populations and numerous unique TCRs (i.e., singletons, Fig. 1). Using SCC3 (Fig. 1a) and TSCC5 (Fig. 1b) as examples, SCCs demonstrated a greater degree of clonal expansion. In SCC3, the top ten clonally expanded TCR sequences accounted for ~31% of TCRs sequenced. Moreover, the top four clonally expanded TCRs accounted for the entire upper quintile of scVDJ reads (ranked by clonal expansion, upper table). In contrast, the top ten clonally expanded TCR sequences of TSCC5 accounted for only ~10% of TCRs sequenced. Additionally, there were twenty-seven unique TCR sequences represented in the upper quintile of scVDJ reads (ranked by clonal expansion, lower table). Globally, more TCR clonotypes were observed in immunocompetent patients (mean = 1140) compared with immunocompromised patients (mean = 544) (*p* < 0.05) (Supplementary Table 2). These findings may be explained by the fact that immunocompromised patients had fewer singletons infiltrating the tumor microenvironment, and TILs present were not expanding in response to local antigens. The majority of TCR sequences recognized putative “known” antigens, including tumor antigens, in both SCC and TSCC samples. Interestingly, up to 24% of TCR sequences from SCC and 22% from TSCC correspond to putative neoantigens (Supplementary Tables 3 and 4).

### cSCC tumors from solid organ transplant patients exhibit notable differences in T cell subpopulations

**Fig. 2:**
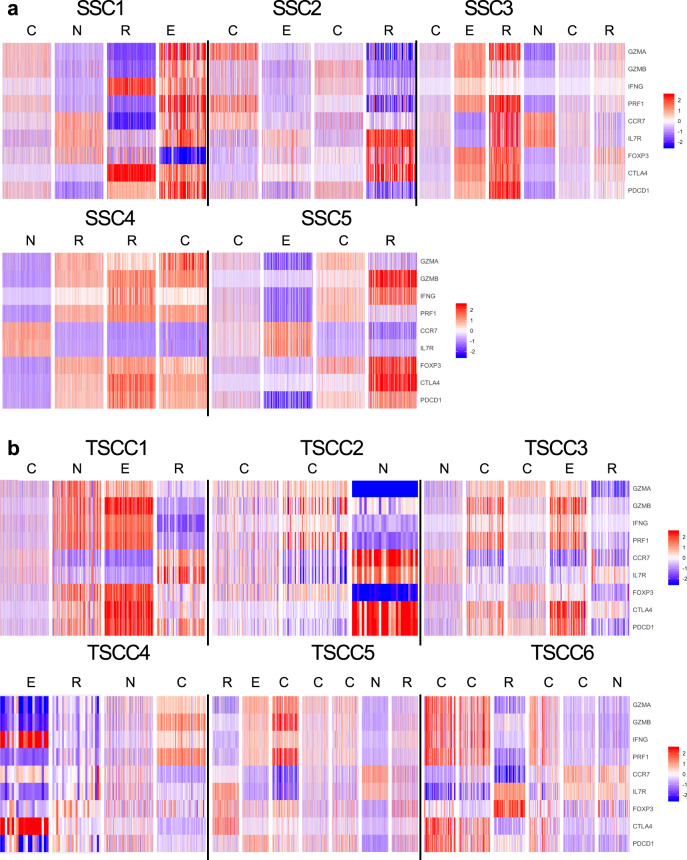
RNA seq derived CD8+TIL phenotyping. Heatmap plots of CD8+T cell RNA expression in SCC (**a**) and TSCC (**b**) tumors reveals distribution of T cell subpopulations. Red indicates increased expression, whereas blue indicates reduced expression. Upper panel shows SCC with naïve (N), cytotoxic (C), regulatory (R), and exhausted (E) subtypes of CD8+TIL. Lower panel shows heatmaps from TSCC with naïve (N), cytotoxic (C), regulatory (R), and exhausted (E).

**Fig. 3:**
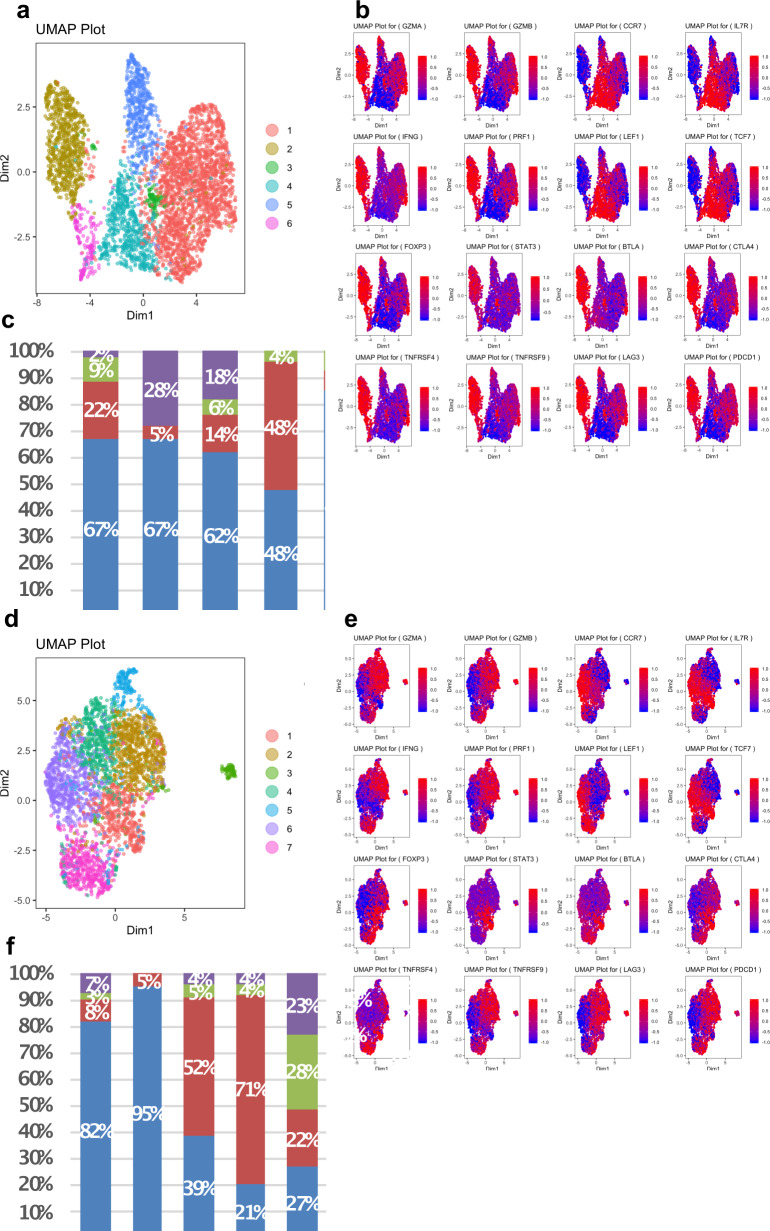
UMAPs and gene expression heat maps for CD8+T lymphocytes from human SCC3 (upper panel) and TSCC5 (lower panel). CD8+tumor infiltrating lymphocytes (*n* = 34,399) obtained from fresh SCC tumor specimens from five immunocompetent patients and CD8+TILs (*n* = 14,902) obtained from fresh TSCC tumor specimens from five immune suppressed transplant patients with six TSCCs were subject to single-cell RNA profiling linked with T-cell receptor (TCR) sequencing. Data were analyzed using iCellR, a custom R package we developed for single cell sequencing analysis. **a**, **d** T cells were clustered according to their top 500 gene expression levels by RNAseq. **b**, **e** Expression of characteristic T lymphocyte genes are shown (clockwise from top left) for cytotoxic, naïve, exhausted and regulatory markers. Red indicates increased expression, whereas blue indicates reduced expression. **c**, **f** Percent proportion of cytotoxic, naïve, regulatory, and exhausted T cell compartment for all tumors and mean of SCC and TSCC tumors.

Single-cell RNA data were used to classify T cells into four subpopulations based on expression of well-characterized markers: cytotoxic (PRF1, GZMA, GZMB, and IFN-γ); naïve (CCR7, LEF1, TCF7, and IL7R); exhausted (BTLA, CTLA4, PDCD1, and LAG3); and regulatory (FOXP3, STAT3, TNFRSF4, and TNFRSF9). Gene expression of T cells from immunocompetent patients reveals naïve, regulatory, cytotoxic or exhausted T cell subpopulations (Fig. 2). Consistent with the administration of immunosuppressive therapy, OTRs generally exhibited lower levels of CD8+TILs (*n* = 6880 immunocompetent SCC; *n* = 2484 immunocompromised SCC, *p* < 0.05). CD8+TILs from TSCCs exhibited more homogeneous gene expression compared with immunocompetent patients. Clustering showed TSCC had lower proportions of cytotoxic (*p* < 0.0001) and naïve (*p* < 0.0001) T cells compared to SCC (Fig. 3, Supplementary Figs. 1–9). Similar numbers of regulatory and exhausted T cells were observed between SCC and TSCC (Fig. 3, Supplementary Figs. 1–9). Of particular note is a clear population of CD8+FOXP3+ regulatory T cells in both SCC and TSCC patients. Traditionally defined CD4+FOXP3+ regulatory TILs have been identified in tumors previously and may play a role in tumor evasion and resistance of the patient’s immune system^10^. However, only recently have a distinct population of CD8+ FOXP3+ regulatory T cells been identified in tumors and never before in cSCC^11^. These CD8+ FOXP3+ regulatory T cells may exhibit an even more potent regulatory function^12^. Further study of this regulatory T cell population is necessary and may reveal novel immunotherapeutic avenues for the treatment of cSCC.

### cSCC exhibits heterogeneity in gene expression

Using iCellR, we found gene expression heterogeneity across patients with cSCC in both groups and even found heterogeneity between cSCC in a given patient. This variable population size is not unprecedented in cancer^13–17^ but has not been described for cSCC. This variation may explain, in part, the otherwise unpredictable level of responses of these tumors to checkpoint inhibition therapy. While some cSCC tumors show complete response to pembrolizumab therapy^18^, other locally advanced or metastatic tumors continue to progress despite such therapy. In our series, TSCC2 and TSCC3 were obtained from the same patient’s forehead and neck, respectively, both considered to represent typical sun-exposed areas. Of note, these tumors exhibited different T cell subpopulation profiles. TSCC2 was largely composed of cytotoxic T cells, whereas exhausted T cells were the most prevalent group in TSCC3, underscoring the importance of local immune response in the progression of and therapeutic outcomes relating to cSCC.

### TCR clonotypes in cSCC recognize putative antigens and neoantigens

**Fig. 4:**
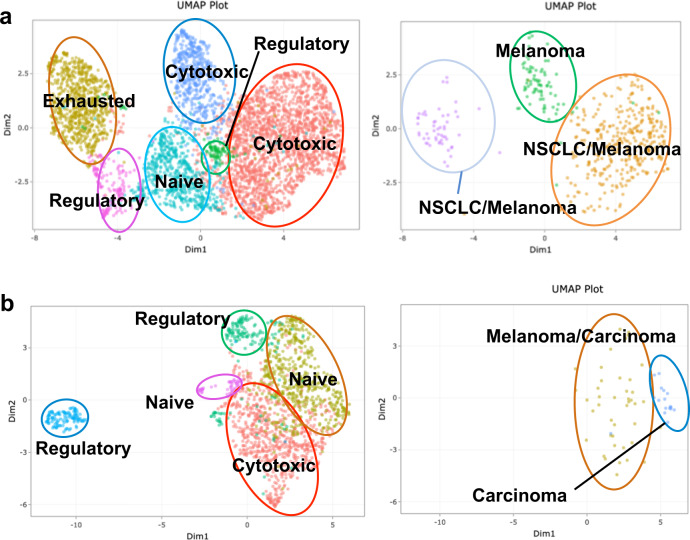
Simultaneous determination of T cell phenotype and TCR specificity using iCellR. **a** UMAP of SCC3 T cell clusters annotated with T cell subpopulation based on gene expression (left) and based on highly expanded TCR clones (right) Number in parenthesis represents overall frequency rank of that epitope from all epitopes in SCC3. **b** UMAP of TSCC3 T cell clusters annotated with T cell subpopulation based on gene expression (left) and highly expanded TCR clones (right). Number in parentheses on TCR clone plots represents rank of expanded clonotype among all clonotypes found in that tumor.

iCellR is able to link the specific TCR sequence of a T cell with that T cell’s gene expression profile via barcode matching. This allowed us to correlate expanded receptor clonotypes with their expected phenotypic function. McPAS-TCR, a manually curated database of pathological antigens associated with different TCRs^19^, was employed to identify putative antigens for these TCR clonotypes. Here, SSC3 is used as an example. Clonotype 5 (green) is associated with melanoma. T cells within this clonotype are generally characterized as cytotoxic T cells. Clonotype 2 (brown) is associated with melanoma and non-small cell lung carcinoma. T cells with this clonotype are generally characterized as cytotoxic T cells (Fig. 4, upper panel, Supplementary Table 4).

Correlation of receptor clonotypes and T cell phenotypes was also performed on tumors from OTRs. However, these clonal T cells do not localize neatly into single T cell phenotypes as do T cells from SCC. For example, two of the top ten expanded clonotypes in the TSCC3 tumor are associated with cancer. Clonotype 9 (blue) is associated with a carcinoma antigen. T cells with this clonotype are generally characterized as naive T cells. However, in other cases, the clonotype spans across multiple phenotypic populations. Clonotype 3 (brown) is common to melanoma. T cells with this clonotype are characterized as either naïve or cytotoxic T cells (Fig. 4, lower panel, Supplementary Table 4). Although beyond the scope of this manuscript, identification and translational application of cSCC neoantigens is an active area of current research by our group which is further enabled by iCellR. We will continue to develop and use our method not only to define TIL landscape but eventually to predict response to checkpoint inhibitor treatment.

We found that SCC from OTRs generally exhibited significantly exhibited lower levels of CD8+ TILs compared to SCC from immune competent patients. Our isolation technique is based on methods we have used extensively^6,20,21^. Variance in numbers of TILs recovered would be expected at the human interface. Moreover, alterations in the local immune microenvironment has been reported to influence outcome in ovarian carcinoma^22^, colorectal carcinoma^16^ and cervical carcinoma^23^.

### Information on T-cell landscape could be potentially used in tumor staging

We defined our study to include only AJCC Stage 2 and BWH T2A tumors. These tumors were >2 cm in diameter and lacked multiple highest risk features, including perineural invasion poor cellular differentiation and invasion beyond fat. We acknowledge that clinical staging systems for cSCC are imperfect^24^. The BWH T staging was established by Schmults and colleagues to address deficiencies in T2 risk stratification by AJCC^25^. Gonzalez et al. showed increased numbers of poor outcomes in low T stage in immune suppressed patients^26^. Blechman et al. showed improved staging for immune suppressed patients using AJCC 8^27^. AJCC 8 included further subdivision of AJCC T2 tumors to T2 and T3 based on the presence of highest risk factors (perineural invasion, poor differentiation, and deep extension) delineated by BWH. In prior studies, we showed distinct MMP profiles for invasive SCC^28^. Some advocate for inclusion of high-risk molecular factors in T staging and prognostication. Our work supports this from the perspective of tumor immunity.

### cSCC tumors from solid organ transplant patients exhibit reduced T effector cells, Tc/Treg ratio and clonality

Despite heterogeneity between patients, and even between sites within one TSCC patient, increased numbers (> 6-fold) of primary cSCC tumors were noted in TSCC patients who as a group showed decreased CD8+ T effector cells, decreased Tc/Treg ratio, and decreased TCR antigen specific clonality. This fold difference approached 10:1 for TSCC patients with catastrophic carcinomatosis defined as >10 SCC per year, extensive field disease, or metastatic event^9^. However, it should be noted that our patient dataset exhibited variables that were not controlled for that could contribute to these differences (i.e., age, gender, and anatomic site). Thus, future work will seek to expand our patient population to further assess the role of these variables in defining the T cell landscape. Other limitations of our study include a relatively small sample size. As these novel techniques become more frequently utilized, cost may decrease and large-scale trials may become feasible. However, Zheng et al. defined a potential role for laylin via single cell sequencing of T cells in from hepatocellular carcinoma with a cohort of six patients^17^. Moreover, based on the small number of patients, no conclusions could be drawn linking disease severity with specific immune suppressive agents. Consistent with our prior work, catastrophic disease was associated with increased length of immune suppression (~25 vs. 7 years, Supplementary Table 1)^29^. Also, of note, there were no metastases in our TSCC patients despite increased numbers of primary SCC. This finding is consistent with our work^27,30^ and others^31,32^ and may be due heightened surveillance, prompt diagnosis and appropriate treatment of cSCC in this high-risk cohort.

We developed a method to define TCR sequence and TIL phenotype simultaneously and found decreased Tc/ Treg ratio in immunosuppressed solid organ transplant recipients. It is likely that heterogeneity extends to other potentially translationally relevant areas, including checkpoint molecule expression and response to neoantigens. We will continue to develop our methods and analytics with the goal of predicting potential response to therapy as well as immune-mediated adverse events.

## Methods

### Ethics of study design and consent

IRB approval for study design was provided by the New York University School of Medicine under Protocol 16-00122. All patients provided informed written consent prior to tumor samples being used for research purposes. The study was performed with strict adherence to the Declaration of Helsinki Principles.

### Single cell suspension derived from patient tumors

Patient tumors were obtained on the day of surgery and washed in cold 1× PBS. Any fat was removed from tumor samples using a scalpel. Then, tumors were then cut into small pieces using a razor and resuspended in 10 mL of RPMI [Gibco 11875-093] supplemented with 5% human serum [Sigma H4522-100], 5 mg gentamicin [Sigma H0887], 5 mg/mL collagenase II [Sigma C2674] and 10 U DNase I [Sigma 00453869] for 10 min at 37 °C. After incubation, the suspension was vortexed 1× for 30 s followed by pipetting sequentially through 25, 10, and 5 mL pipettes for 1 min each. Next, the cell suspension was filtered through a 70-μm filter [Fisher 22363548] and spun at 450 g for 10 min. After spinning, the supernatant was removed, and ACK red blood cell lysis was performed according to the manufacturer’s protocol as needed [Gibco a10492-01]. Cells were then stained as described below prior to cell sorting and FACS analysis.

### Flow cytometry

Approximately 1.0 to 5.0 × 10^6^ cells were resuspended in FACS buffer [2% FBS in 1× PBS] and stained with the live/dead Aqua cell stain kit [Invitrogen L34957] for 30 min on ice. Cells were then washed 1× with FACS buffer and blocked with 1:50 dilution of Human Fc block [Biolegend 422302] for 15 min on ice. Afterwards, the cells were washed with FACS buffer followed by staining with PE anti-human TCRα/β (1:200 dilution) [Biolegend 306707], PECY7 anti-human CD4 (1:200 dilution) [Biolegend 300511], and APC anti-human CD8a (1:50 dilution) [Biolegend 301014] for 25 min on ice followed by washing with 1× FACS buffer and filtering using a 50-µm filter [Sysmex 04-004-2327] prior to analysis. All cell sorting and FACS analysis were performed using BD FACSaria II.

### Single-cell library construction and sequencing- 5′ and VDJ

The sorted cellular suspensions were loaded on a 10× Genomics Chromium instrument to generate single-cell gel beads in emulsion (GEMs). Approximately 10–12e3 cells were loaded per channel. Single-cell RNA-Seq libraries were prepared using the following single cell 5′ reagent kits: Chromium™ single cell 5′ Library & Gel Bead Kit, PN-1000006 and chromium single cell VDJ enrichment kit for human T cells (PN-100000). Libraries were run on a NovaSeq 6000 SP or S1 flow cell (depending on sample numbers) for both 5′ transcriptome data and TCR clonotype determination.

### Alignment, barcode assignment, and UMI counting

The Cell Ranger for VDJ software suite was used for analysis. It consists of a series of analysis pipelines that process Chromium single cell 5′ RNA-seq output to assemble, quantify, and annotate paired VDJ transcript sequences. Cell Ranger includes two pipelines specific to VDJ analysis, though integrated experiments may also make use of pipelines for gene expression, especially cellranger count.

cellranger mkfastq demultiplexes raw base call files generated by Illumina sequencers into FASTQ files. It is a wrapper around Illumina’s bcl2fastq, with additional useful features that are specific to 10× libraries and a simplified sample sheet format. (1) cellranger vdj takes FASTQ files from cellranger mkfastq and performs VDJ sequence assembly and paired clonotype calling. It uses the Chromium cellular barcodes and UMIs to assemble V(D)J transcripts cell-by-cell. cellranger can take input from multiple sequencing runs on the same library. (2) cellranger count can, as of version 2.1 or greater, perform gene expression analysis on 5′ sequencing data. (See Single Cell V(D)J+5′ Gene Expression for more details.) Output is delivered in standard BAM, CSV, FASTA, FASTQ, JSON, and HTML formats that are augmented with cell and clonotype-specific information.

### Bioinformatics analysis

Bioinformatics analysis was based on methods validated in previous studies^33^. Specifically, after confirming the integrity of the cDNA, quality of the libraries, number of cells sequenced and mean number of reads per cell, as a quality control, we used the cellranger package to map the reads and generate gene-cell matrices. A quality control was then performed on the cells to calculate the number of genes, UMIs and the proportion of mitochondrial genes for each cell using iCellR R package (v1) (https://github.com/rezakj/iCellR) and the cells with low number of covered genes (gene count < 500) and high mitochondrial counts (mt-genes > 0.1) were filtered out. Then, the matrix was normalized based on ranked geometric library size factor (ranked glsf) using iCellR. Geometric library size factor normalization is a common normalization method used by popular tools such as DEseq2; however, here we use only the top ranked genes (top 500 genes sorted by base mean), this is for reducing the effect of dropouts (nonzero events counted as zero) in normalization by taking into account only the highly expressed genes. A general gene statistics analysis was then performed to calculate gene dispersion, base mean and cell coverage to use to build a gene model for performing principal component analysis (PCA). Genes with high coverage (top 500) and high dispersion (dispersion >1.5) were chosen and PCA analysis was performed, a second round of PCA was performed based on the top 20 and bottom 20 genes predicted in the first ten dimensions of PCA to fine tune the results and then the clustering was performed (iCellR options; clust.method = “kmeans”, dist.method = “euclidean”, index.method = “silhouette”) on the principal components with high standard deviation (top ten PCs) and T-distributed stochastic neighbor embedding was performed. Uniform manifold approximation and projection (UMAP) was also performed on the top ten PCs. Marker genes for each cluster were determined based on fold-change and adjusted *p*-value (*t*-test), and average gene expression for each cluster was calculated using iCellR. Marker genes were visualized on heatmaps, bar plots, and box plots for each cluster and were used to assign cell phenotypes to each cluster. Finally, the VDJ sequencing data and clonotypes were visualized on the dimensionality reduction maps (tSNE and UMAP) and their frequency was calculated to assign clones to different phenotypic clusters. Additionally, the McPAS-TCR database was utilized to query CDR3 α/β T cell chains for antigen matches to those previously described, stratified and ranked by Levenshtein distance (http://friedmanlab.weizmann.ac.il/McPAS-TCR).

### Reporting summary

Further information on research design is available in the Nature Research Reporting Summary linked to this article.

## Supplementary information

Supplementary Figures and Tables

Reporting Summary

## Data Availability

All data, including single-cell RNAseq and TCR sequencing, are available from the NCBI GEO Database under accession number GSE145328.

## Code availability

iCellR R package (v1) is available at (https://github.com/rezakj/iCellR).
