# Supplementary material for: Decreased cytotoxic T cells and TCR clonality in organ transplant recipients with squamous cell carcinoma
Source: NPJ Precis Oncol. 2020 Jun 3;4:13. doi: 10.1038/s41698-020-0119-9 (PMC7270180; doi:10.1038/s41698-020-0119-9)
Supplement: Supplementary file 1 — Supplementary Figures and Tables [file 41698_2020_119_MOESM1_ESM.pdf]

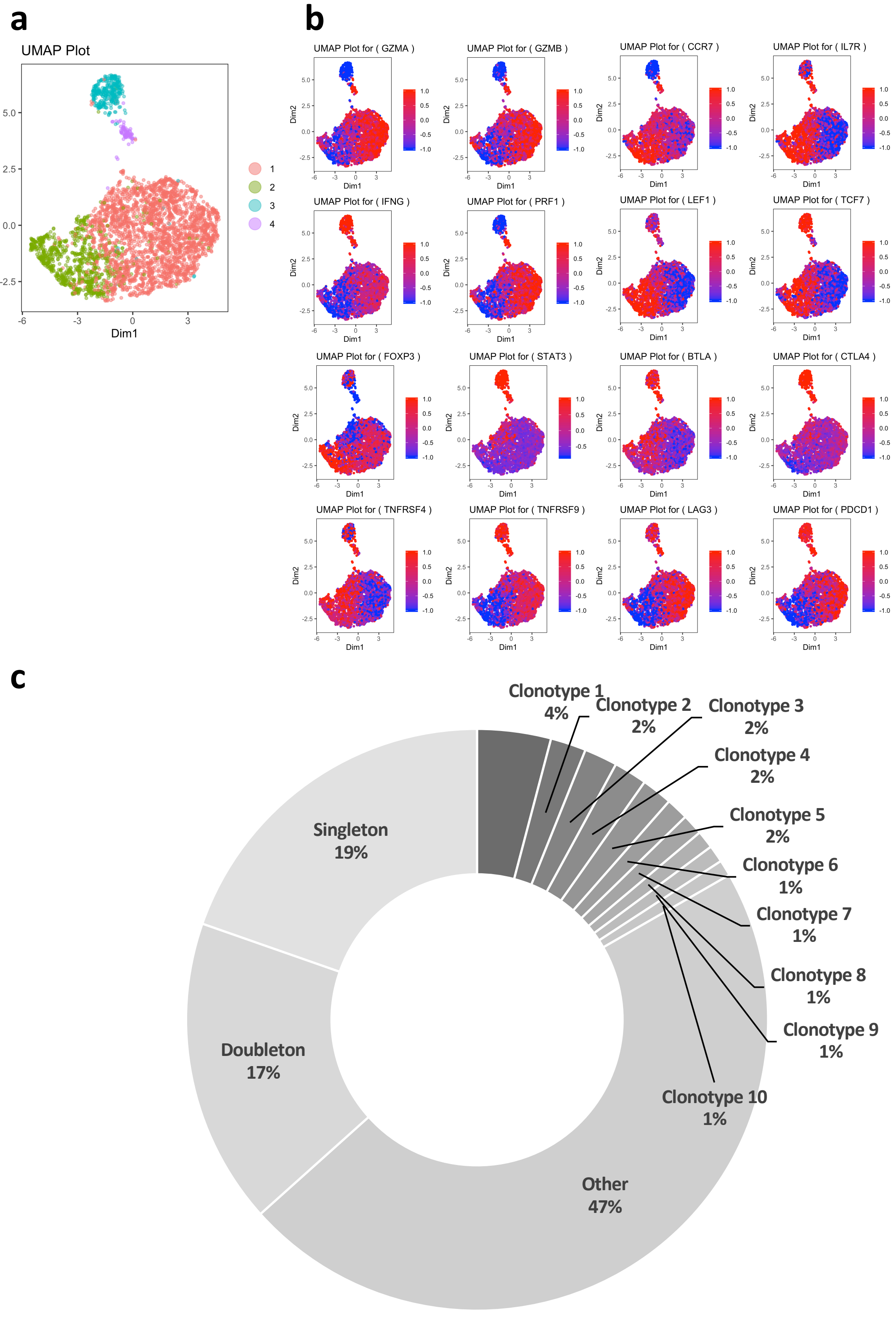

Number of Unique TCR Clonotypes Per Quintile of Total scVDJ Reads	
16	Upper Quintile
50	Uppermid Quintile
153	Middle Quintile
321	Lowermid Quintile
756	Lower Quintile

Supplementary Figure 1. UMAPs and gene expression heat maps for CD8+ T lymphocytes from human SCC1. CD8+ tumor infiltrating lymphocytes obtained from fresh SCC tumor specimens from 5 immunocompetent patients were subject to single-cell RNA profiling linked with T-cell receptor (TCR) sequencing. Data were analyzed using iCellR, a custom R package we developed for single cell sequencing analysis. (a) T cells were clustered according to their top 500 gene expression levels by RNAseq. (b) Expression of characteristic T lymphocyte genes are shown (clockwise from top left) for cytotoxic, naïve, exhausted and regulatory markers. Red indicates increased expression, whereas blue indicates reduced expression. (c) Percent of total scVDJ sequencing reads represented by unique clonotypes. Clonotypes 1-10 are the most frequently appearing clonotypes in SCC1. Doubletons are all clonotypes appearing twice and singletons are all clonotypes appearing once in SCC1. Other is all tripletons or higher, excluding the top ten most frequent clonotypes.

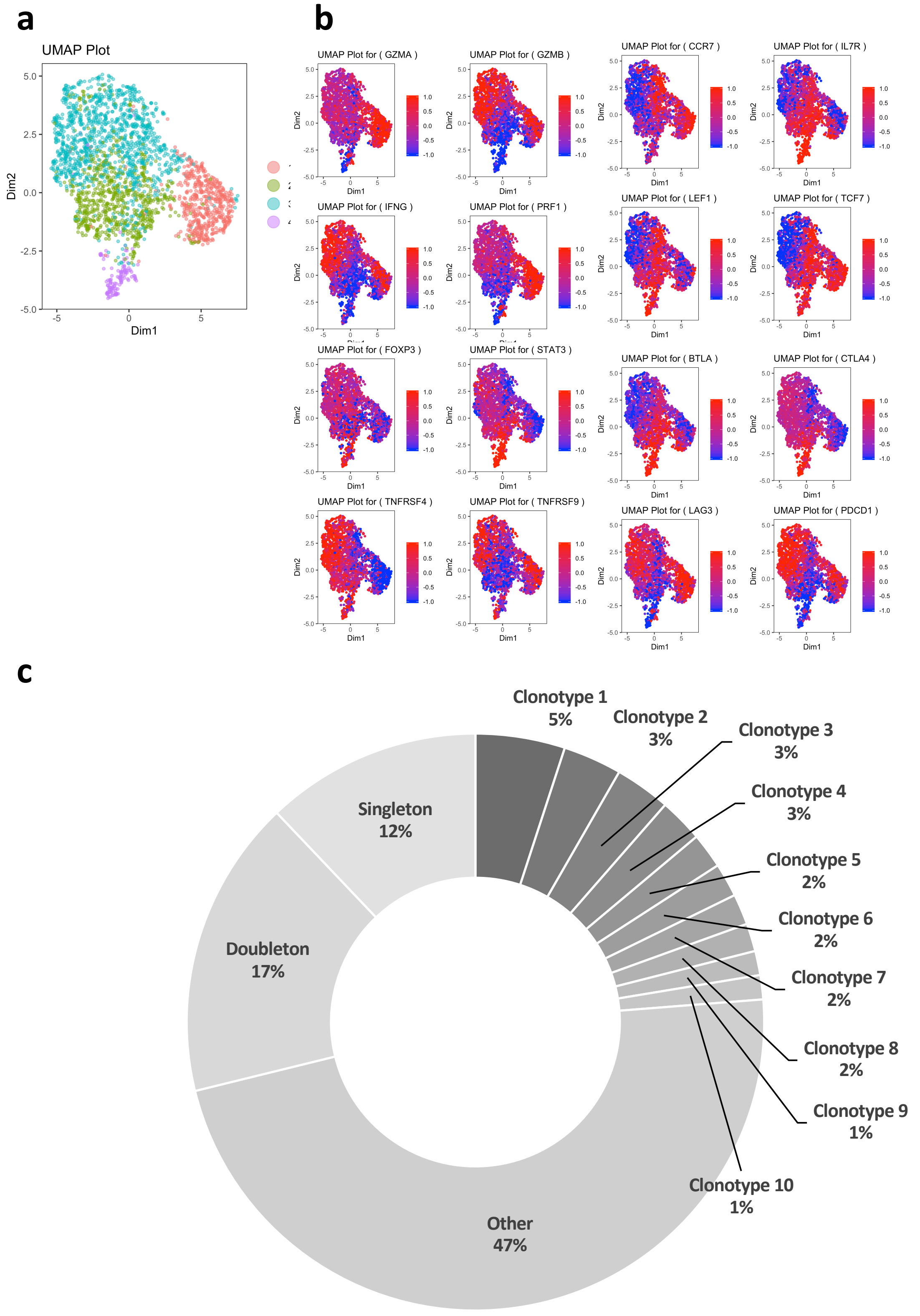

Number of Unique TCR Clonotypes Per Quintile of Total scVDJ Reads	
8	Upper Quintile
25	Uppermid Quintile
69	Middle Quintile
195	Lowermid Quintile
515	Lower Quintile

Supplementary Figure 2. UMAPs and gene expression heat maps for CD8+ T lymphocytes from human SCC2. CD8+ tumor infiltrating lymphocytes obtained from fresh SCC tumor specimens from 5 immunocompetent patients were subject to single-cell RNA profiling linked with T-cell receptor (TCR) sequencing. Data were analyzed using iCellR, a custom R package we developed for single cell sequencing analysis. (a) T cells were clustered according to their top 500 gene expression levels by RNAseq. (b) Expression of characteristic T lymphocyte genes are shown (clockwise from top left) for cytotoxic, naïve, exhausted and regulatory markers. Red indicates increased expression, whereas blue indicates reduced expression. (c) Percent of total scVDJ sequencing reads represented by unique clonotypes. Clonotypes 1-10 are the most frequently appearing clonotypes in SCC2. Doubletons are all clonotypes appearing twice and singletons are all clonotypes appearing once in SCC2. Other is all tripletons or higher, excluding the top ten most frequent clonotypes.

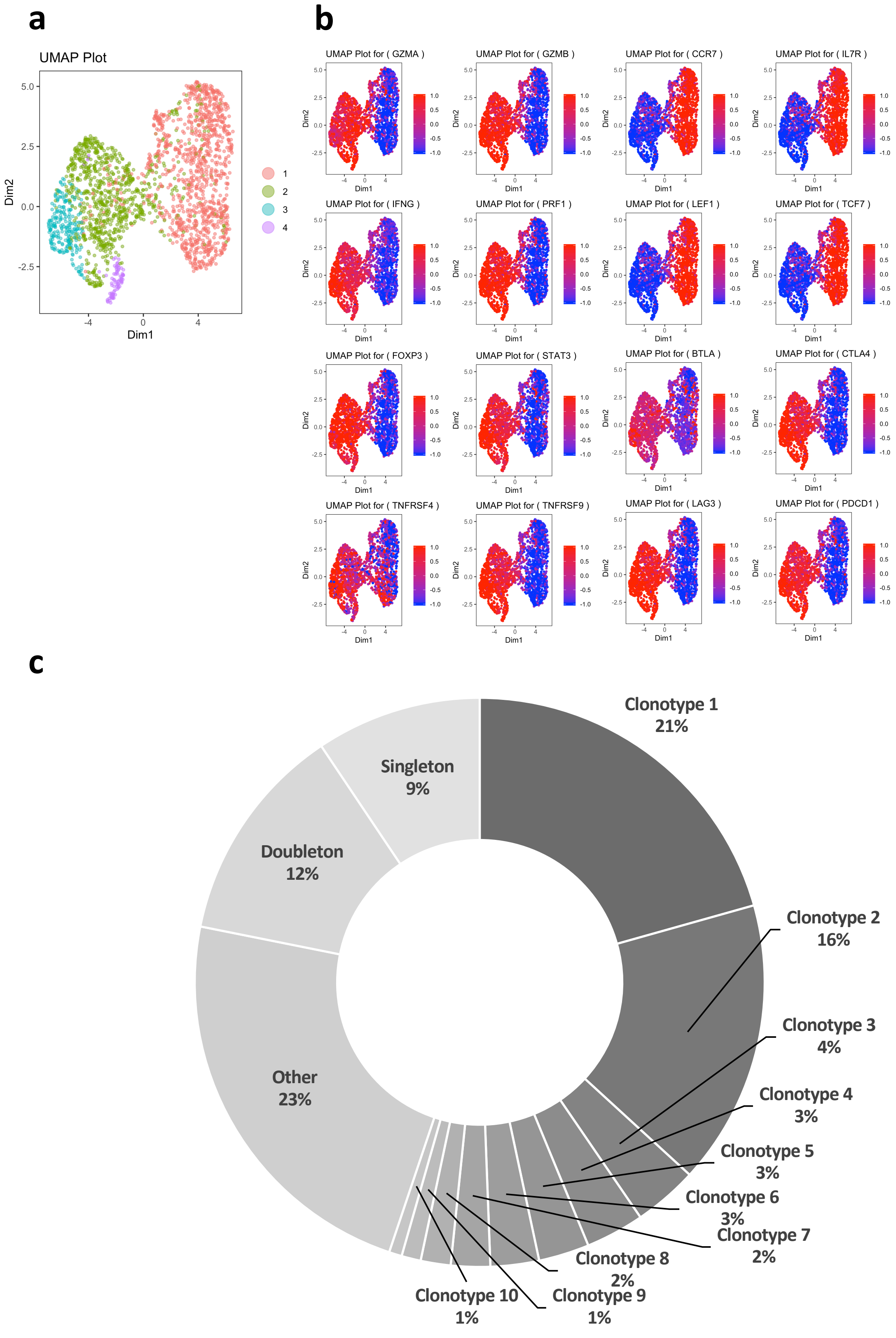

Number of Unique TCR Clonotypes Per Quintile of Total scVDJ Reads	
1	Upper Quintile
2	Uppermid Quintile
16	Middle Quintile
107	Lowermid Quintile
409	Lower Quintile

Supplementary Figure 3. UMAPs and gene expression heat maps for CD8+ T lymphocytes from human SCC4. CD8+ tumor infiltrating lymphocytes obtained from fresh SCC tumor specimens from 5 immunocompetent patients were subject to single-cell RNA profiling linked with T-cell receptor (TCR) sequencing. Data were analyzed using iCellR, a custom R package we developed for single cell sequencing analysis. (a) T cells were clustered according to their top 500 gene expression levels by RNAseq. (b) Expression of characteristic T lymphocyte genes are shown (clockwise from top left) for cytotoxic, naïve, exhausted and regulatory markers. Red indicates increased expression, whereas blue indicates reduced expression. (c) Percent of total scVDJ sequencing reads represented by unique clonotypes. Clonotypes 1-10 are the most frequently appearing clonotypes in SCC4. Doubletons are all clonotypes appearing twice and singletons are all clonotypes appearing once in SCC4. Other is all tripletons or higher, excluding the top ten most frequent clonotypes.

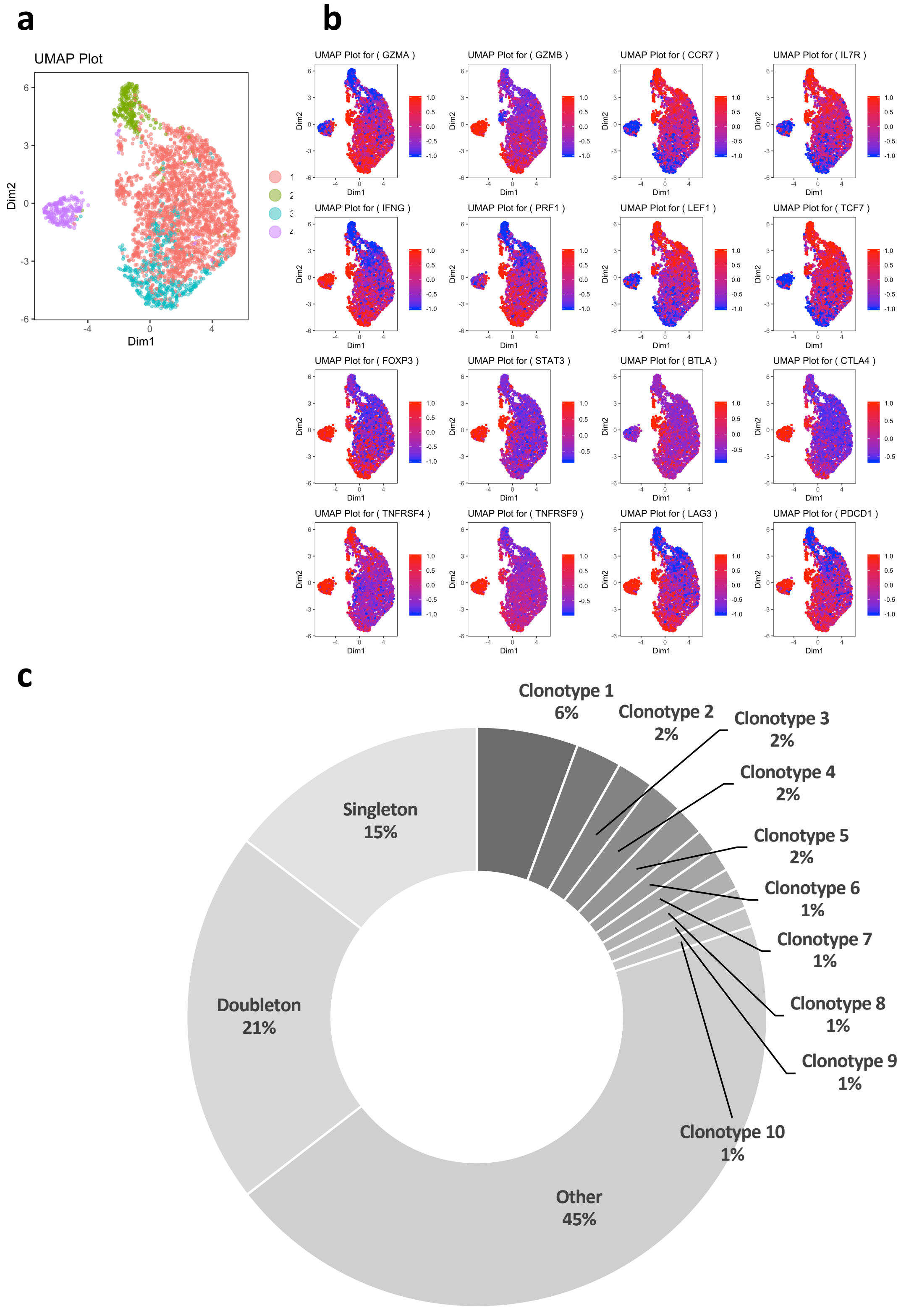

Number of Unique TCR Clonotypes Per Quintile of Total scVDJ Reads	
11	Upper Quintile
43	Uppermid Quintile
123	Middle Quintile
332	Lowermid Quintile
789	Lower Quintile

Supplementary Figure 4. UMAPs and gene expression heat maps for CD8+ T lymphocytes from human SCC5. CD8+ tumor infiltrating lymphocytes obtained from fresh SCC tumor specimens from 5 immunocompetent patients were subject to single-cell RNA profiling linked with T-cell receptor (TCR) sequencing. Data were analyzed using iCellR, a custom R package we developed for single cell sequencing analysis. (a) T cells were clustered according to their top 500 gene expression levels by RNAseq. (b) Expression of characteristic T lymphocyte genes are shown (clockwise from top left) for cytotoxic, naïve, exhausted and regulatory markers. Red indicates increased expression, whereas blue indicates reduced expression. (c) Percent of total scVDJ sequencing reads represented by unique clonotypes. Clonotypes 1-10 are the most frequently appearing clonotypes in SCC5. Doubletons are all clonotypes appearing twice and singletons are all clonotypes appearing once in SCC5. Other is all tripletons or higher, excluding the top ten most frequent clonotypes.

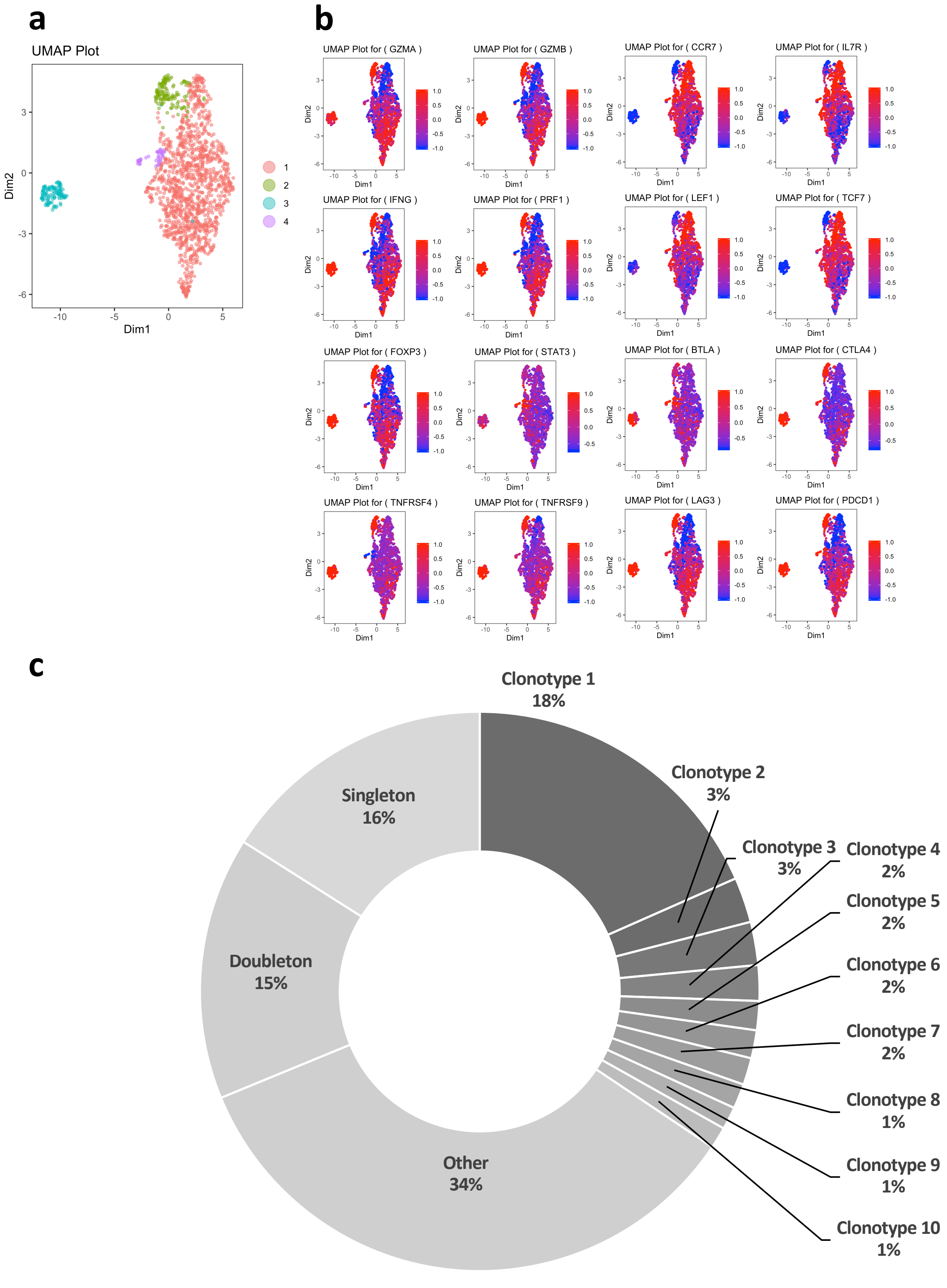

Number of Unique TCR Clonotypes Per Quintile of Total scVDJ Reads	
2	Upper Quintile
14	Uppermid Quintile
46	Middle Quintile
136	Lowermid Quintile
354	Lower Quintile

Supplementary Figure 5. UMAPs and gene expression heat maps for CD8+ T lymphocytes from human TSCC1. CD8+ tumor infiltrating lymphocytes obtained from fresh TSCC tumor specimens from 5 immune suppressed transplant patients with 6 TSCCs were subject to single-cell RNA profiling linked with T-cell receptor (TCR) sequencing. Data were analyzed using iCellR, a custom R package we developed for single cell sequencing analysis. (a) T cells were clustered according to their top 500 gene expression levels by RNAseq. (b) Expression of characteristic T lymphocyte genes are shown (clockwise from top left) for cytotoxic, naïve, exhausted and regulatory markers. Red indicates increased expression, whereas blue indicates reduced expression. (c) Percent of total scVDJ sequencing reads represented by unique clonotypes. Clonotypes 1-10 are the most frequently appearing clonotypes in TSCC1. Doubletons are all clonotypes appearing twice and singletons are all clonotypes appearing once in TSCC1. Other is all tripletons or higher, excluding the top ten most frequent clonotypes.

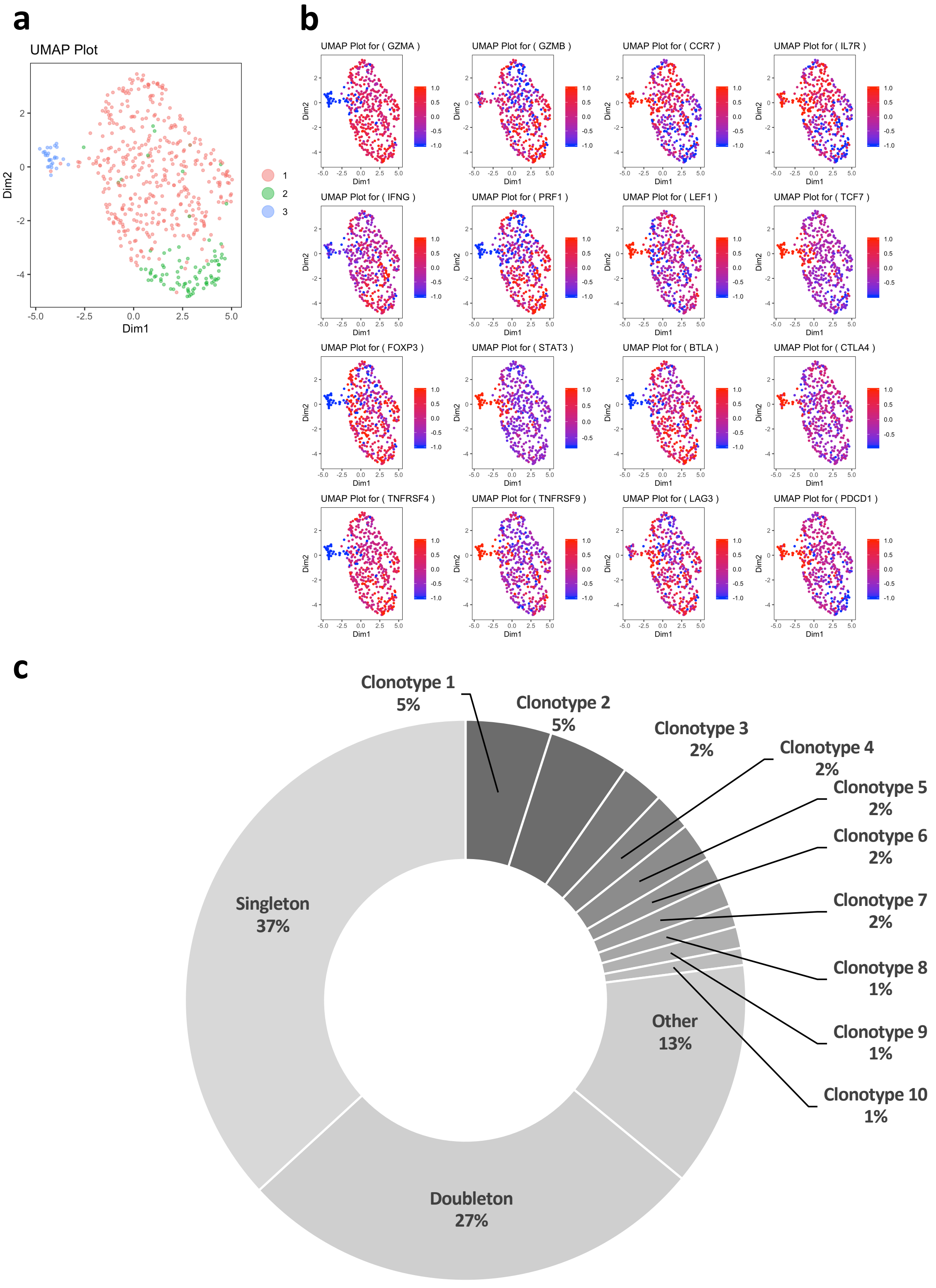

Number of Unique TCR Clonotypes Per Quintile of Total scVDJ Reads	
8	Upper Quintile
29	Uppermid Quintile
42	Middle Quintile
80	Lowermid Quintile
120	Lower Quintile

Supplementary Figure 6. UMAPs and gene expression heat maps for CD8+ T lymphocytes from human TSCC2. CD8+ tumor infiltrating lymphocytes obtained from fresh TSCC tumor specimens from 5 immune suppressed transplant patients with 6 TSCCs were subject to single-cell RNA profiling linked with T-cell receptor (TCR) sequencing. Data were analyzed using iCellR, a custom R package we developed for single cell sequencing analysis. (a) T cells were clustered according to their top 500 gene expression levels by RNAseq. (b) Expression of characteristic T lymphocyte genes are shown (clockwise from top left) for cytotoxic, naïve, exhausted and regulatory markers. Red indicates increased expression, whereas blue indicates reduced expression. (c) Percent of total scVDJ sequencing reads represented by unique clonotypes. Clonotypes 1-10 are the most frequently appearing clonotypes in TSCC2. Doubletons are all clonotypes appearing twice and singletons are all clonotypes appearing once in TSCC2. Other is all tripletons or higher, excluding the top ten most frequent clonotypes.

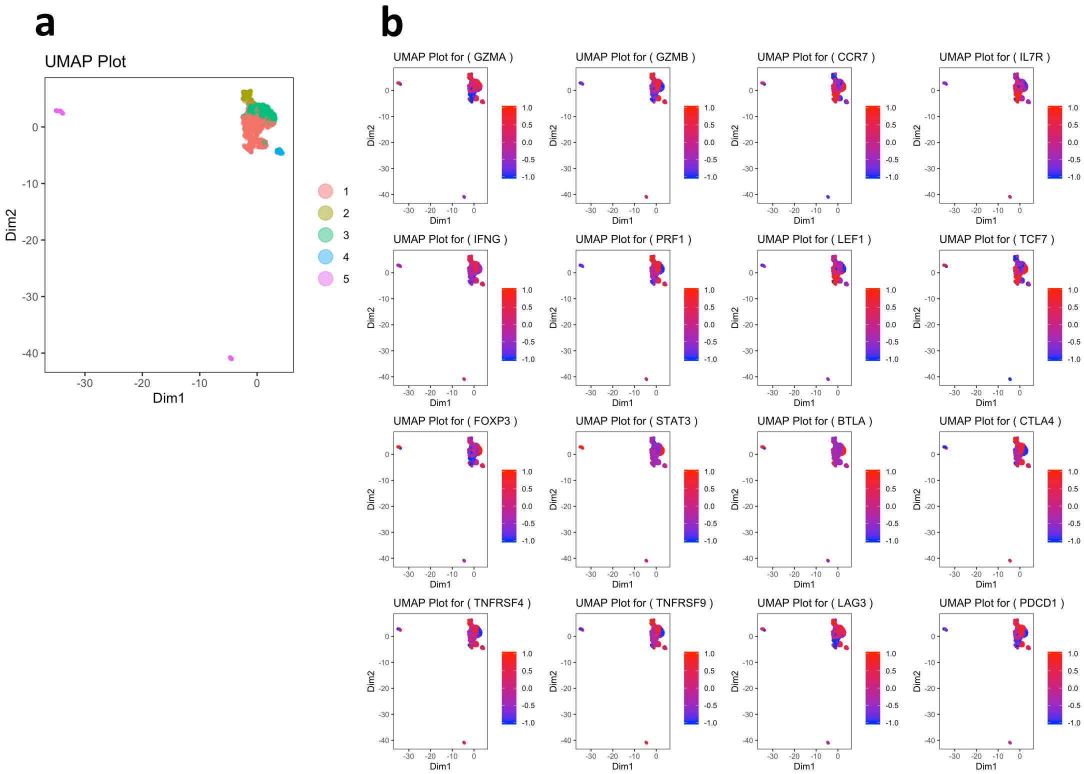

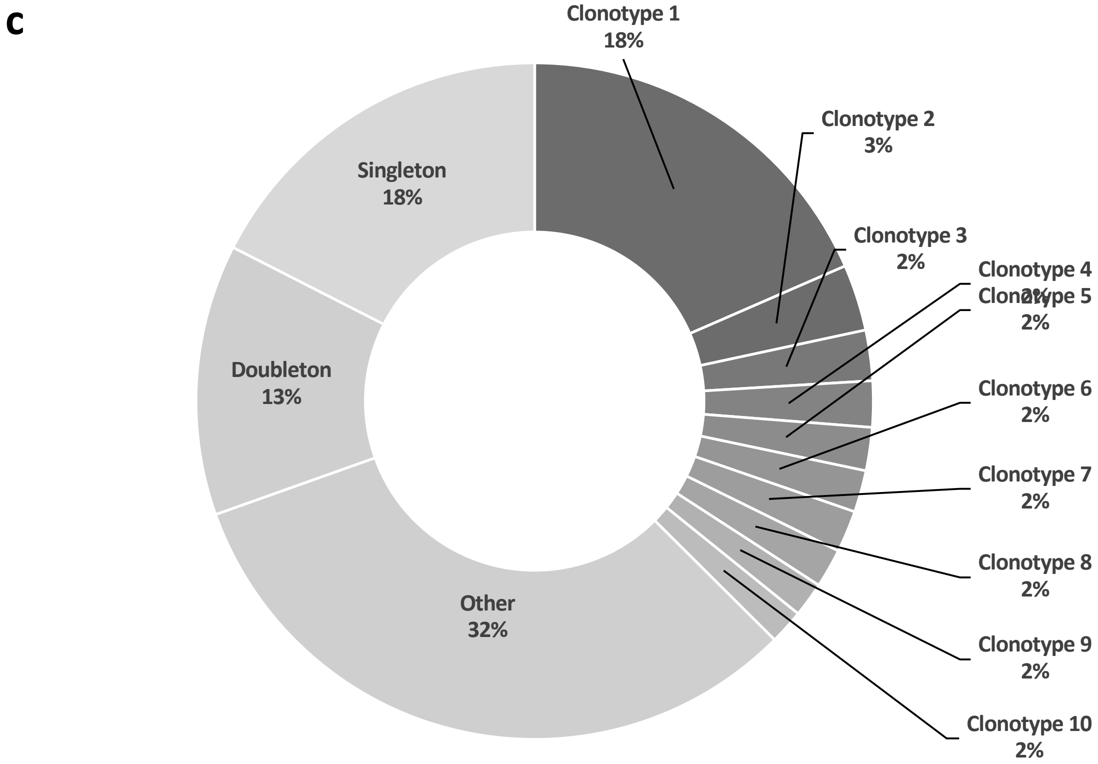

Number of Unique TCR Clonotypes Per Quintile of Total scVDJ Reads	
2	Upper Quintile
11	Uppermid Quintile
33	Middle Quintile
199	Lowermid Quintile
326	Lower Quintile

Supplementary Figure 7. UMAPs and gene expression heat maps for CD8+ T lymphocytes from human TSCC3. CD8+ tumor infiltrating lymphocytes obtained from fresh TSCC tumor specimens from 5 immune suppressed transplant patients with 6 TSCCs were subject to single-cell RNA profiling linked with T-cell receptor (TCR) sequencing. Data were analyzed using iCellR, a custom R package we developed for single cell sequencing analysis. (a) T cells were clustered according to their top 500 gene expression levels by RNAseq. (b) Expression of characteristic T lymphocyte genes are shown (clockwise from top left) for cytotoxic, naïve, exhausted and regulatory markers. Red indicates increased expression, whereas blue indicates reduced expression. (c) Percent of total scVDJ sequencing reads represented by unique clonotypes. Clonotypes 1-10 are the most frequently appearing clonotypes in TSCC3. Doubletons are all clonotypes appearing twice and singletons are all clonotypes appearing once in TSCC3. Other is all tripletons or higher, excluding the top ten most frequent clonotypes.

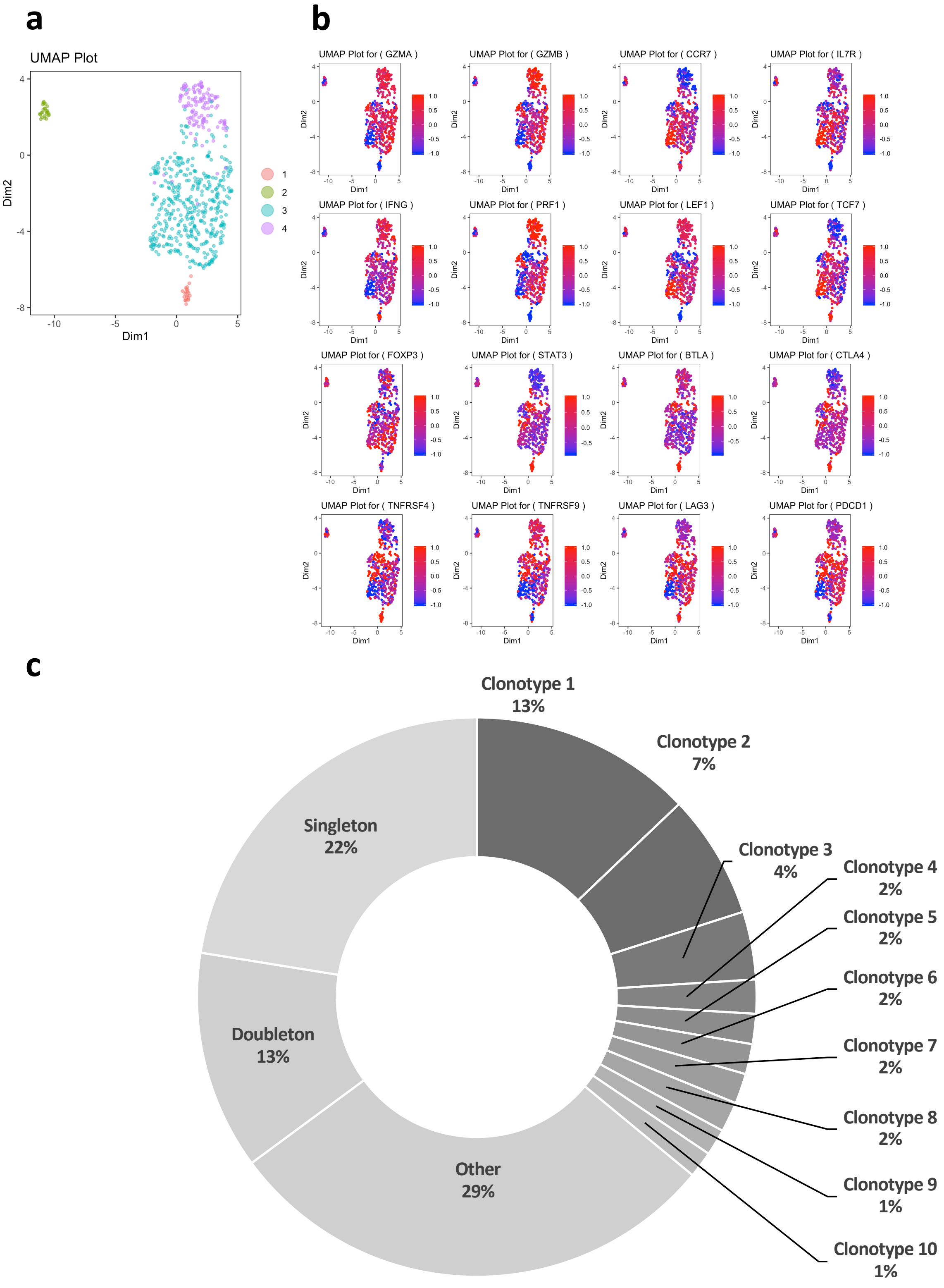

Number of Unique TCR Clonotypes Per Quintile of Total scVDJ Reads	
2	Upper Quintile
13	Uppermid Quintile
25	Middle Quintile
64	Lowermid Quintile
124	Lower Quintile

Supplementary Figure 8. UMAPs and gene expression heat maps for CD8+ T lymphocytes from human TSCC4. CD8+ tumor infiltrating lymphocytes obtained from fresh TSCC tumor specimens from 5 immune suppressed transplant patients with 6 TSCCs were subject to single-cell RNA profiling linked with T-cell receptor (TCR) sequencing. Data were analyzed using iCellR, a custom R package we developed for single cell sequencing analysis. (a) T cells were clustered according to their top 500 gene expression levels by RNAseq. (b) Expression of characteristic T lymphocyte genes are shown (clockwise from top left) for cytotoxic, naïve, exhausted and regulatory markers. Red indicates increased expression, whereas blue indicates reduced expression. (c) Percent of total scVDJ sequencing reads represented by unique clonotypes. Clonotypes 1-10 are the most frequently appearing clonotypes in TSCC4. Doubletons are all clonotypes appearing twice and singletons are all clonotypes appearing once in TSCC4. Other is all tripletons or higher, excluding the top ten most frequent clonotypes.

a

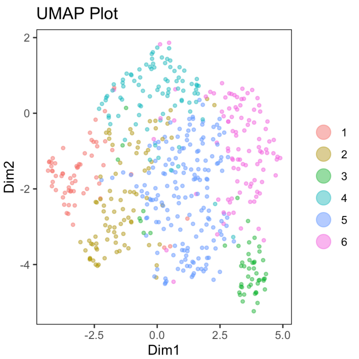

b

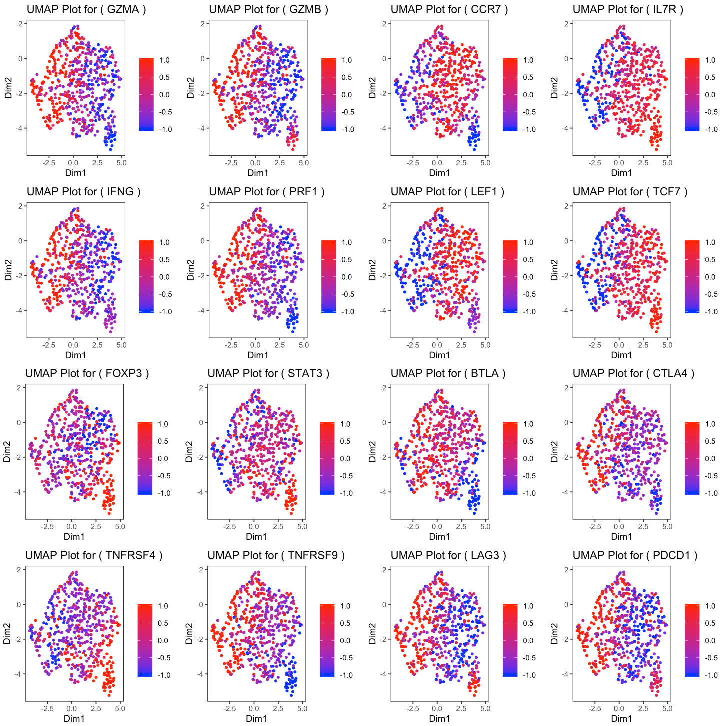

c

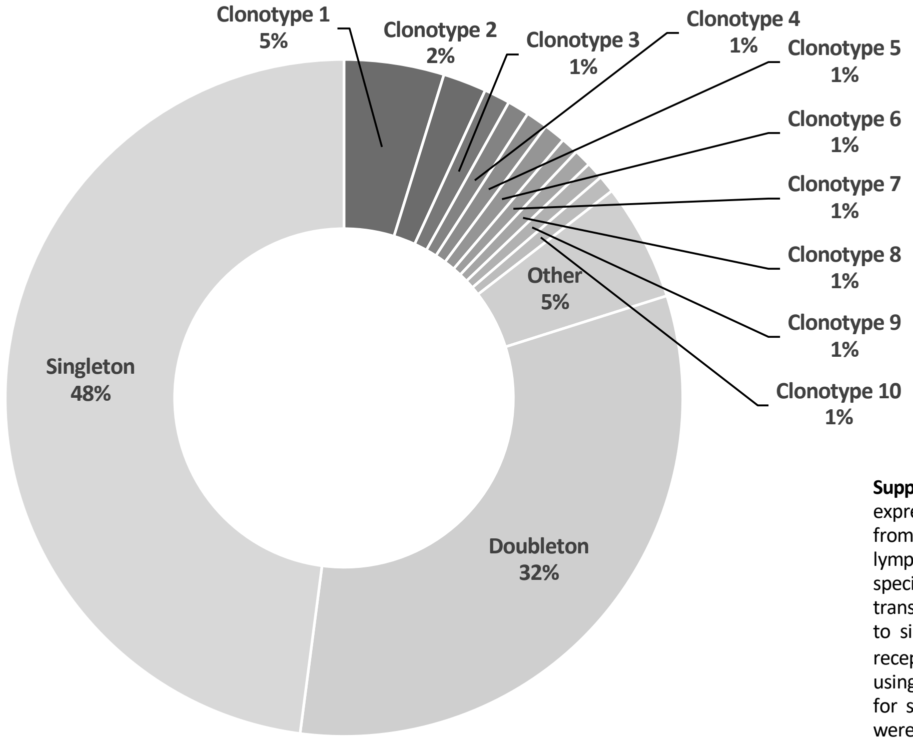

Number of Unique TCR Clonotypes Per Quintile of Total scVDJ Reads	
19	Upper Quintile
48	Uppermid Quintile
67	Middle Quintile
97	Lowermid Quintile
375	Lower Quintile

Supplementary Figure 9. UMAPs and gene expression heat maps for CD8+ T lymphocytes from human TSCC6. CD8+ tumor infiltrating lymphocytes obtained from fresh TSCC tumor specimens from 5 immune suppressed transplant patients with 6 TSCCs were subject to single-cell RNA profiling linked with T-cell receptor (TCR) sequencing. Data were analyzed using iCellR, a custom R package we developed for single cell sequencing analysis. (a) T cells were clustered according to their top 500 gene expression levels by RNAseq. (b) Expression of characteristic T lymphocyte genes are shown (clockwise from top left) for cytotoxic, naïve, exhausted and regulatory markers. Red indicates increased expression, whereas blue indicates reduced expression. (c) Percent of total scVDJ sequencing reads represented by unique clonotypes. Clonotypes 1-10 are the most frequently appearing clonotypes in TSCC6. Doubletons are all clonotypes appearing twice and singletons are all clonotypes appearing once in TSCC6. Other is all tripletons or higher, excluding the top ten most frequent clonotypes.

Supplementary Table 1. Patient and tumor characteristics.

Tumor	Sex	Age	Site	AJCC Stage	BWH Stage	Number of Tumors/ 12 mo	Immunosuppression Regimen	Organ Transplant
SCC1	F	83	Leg	2	2A	1		
SCC2	F	84	Ear	1	1	1		
SCC3	M	86	Leg	2	2A	1		
SCC4	M	94	Forehead	2	2A	1		
SCC5	M	58	Forehead	2	2A	2		
TSCC1	F	71	Leg	2	2A	6	Tacrolimus, mycophenolate	Liver (2009)
TSCC2	F	56	Forehead	2	2A	10	Azathioprine, prednisone	Kidney (1985)
TSCC3	F	56	Neck	2	2A	10	Azathioprine, prednisone	Kidney (1985)
TSCC4	F	80	Foot	2	2A	9	Sirolimus, prednisonme, tacrolimus	Kidney (1986), Liver/Dual Kidney (2004)
TSCC5	M	66	Temple	2	2A	3	Mycophenolate, tacrolimus	Liver (2011)
TSCC6	F	61	Scalp	2	2A	4	Prednisone, mycophenoalte, cyclosporine	Liver/Dual Kidney (2011)

Supplementary Table 2. Total clonotypes and total VDJ sequencing reads in cSCC tumors from immunocompetent (SCC1-5) and immunocompromised (TSCC1-6) patients.

Tumor	Total Clonotypes	Total Reads
SCC1	1292	5856
SCC2	1222	4869
SCC3	1765	11508
SCC4	531	4944
SCC5	1295	7222
TSCC1	549	3210
TSCC2	275	809
TSCC3	488	2904
TSCC4	225	917
TSCC5	1462	6098
TSCC6	375	964

Supplementary Table 3. Percentage of known antigen versus neoantigen among the top 10 clonotypes in cSCC tumors from immunocompetent (SCC1-5) and immunocompromised (TSCC1-6) patients.

Tumor	TCR-alpha Known Antigen	TCR-alpha Neoantigen	TCR-beta Known Antigen	TCR-beta Neoantigen
SCC1	100%	0%	90%	10%
SCC2	68%	32%	90%	10%
SCC3	60%	40%	87%	13%
SCC4	79%	21%	93%	7%
SCC5	74%	26%	96%	4%
Average	76%	24%	91%	9%
TSCC1	68%	32%	88%	13%
TSCC2	96%	4%	91%	9%
TSCC3	82%	18%	89%	11%
TSCC4	80%	20%	92%	8%
TSCC5	67%	33%	89%	12%
TSCC6	75%	25%	91%	9%
Average	78%	22%	90%	10%

Supplementary Table 4. Putative antigens of the top 10 clonotypes in cSCC tumors from immunocompetent (SCC1-5) and immunocompromised (TSCC1-6) patients based on McPAS analysis (wobble < 8).

Sample	Clonotype	Alpha CDR3	Putative Antigen Classification	Putative Antigens	Beta CDR3	Putative Antigen Classification	Putative Antigens
SCC1	1	CAVKDFNKFYF	Cancer	SEC24A-FLY	CASSLFSGTGTNEQFF		
	2	CILRGAGGTSYGKLTF			CASSLQGANYEQYF	Melanoma, Cancer	Mutated CDKNA2, ATP6AP-KLG_G3W, FNDC3B_L3M
	3	CAMKAGGTSYGKLTF	Melanoma	MART-1	CASSPSGRGHEQYF		
	4	CAMRDLYSGAGSYQLTF	Cancer	NSDHL-A9V	CASSLDASGRGYEQYF	Renal Clear Cell Carcinoma	
	5				CASSQDGAGWADQPQHF		
	6				CASSLFSGTGTNEQFF		
	7	CAVRDMSSGNTGKLIF			CASRFDWGRDTEAFF		
	8	CAVRGSGTYKYIF	Cancer	SREBF1-YLQ_L6M	CASSLSKGDGYGYTF	Melanoma	Mutated CDKNA2
	9	CASSPSLRTLTADTQYF			CASSYGHGETQYF		
	10	CLVGDIPRRYNFNKFYF	Cancer	OR14C36-FML_V6L	CASSPTLTGDLTDTQYF		
SCC2	1	CAVLPAYGGSQGNLIF			CASSRGPNPRTEAFF		
	2	CAVSYNDMRF	Neoantigen	LCP1-NLF	CASSPLAGGNLETQYF	Melanoma, Colorectal Cancer	MART-1
	3	CALTRNNNARLMF			CATAGVQETQYF	Neoantigen	PIGN-FLT_P7H
	4	CATDAPDSNYQLIW	Neoantigen	STOX1-RLM_M3I	CASSYKGNNSPLHF	Neoantigen	FNDC3B
	5	CALSDTETSGSRLTF			CASSGTNQETQYF	Melanoma, Neoantigen	Mutated CDKNA2, GANAG-S5F
	6	CAFIDSGAGSYQLTF	Neoantigen	HOXC9-YMY, GNL3L_R4C	CRIPSSTDTQYF		
	7	CAMSFTQYNDMRF			CASSLSGEVRNEQFF		
	8	CAAIPWGNAGKSTF	Neoantigen	GNL3L_R4C	CASSIPVGLGYGYTF	Neoantigen	CNKSR1-SLA_A9V, SEC24A-FLY
	9				CASSRGPNPRTEAFF		
	10	CAVRHISNQFYF	Neoantigen	IPO9-FSS, MLL2-L8H	CASSDRGRSNEQFF		
SCC3	1	CAVNKNYDYKLSF	Neoantigen	CNKSR1-SLA_A9V, DCHS1-TLF_I5M, PHKA2-LLS	CASSLEEGTTDTQYF		
	2	CAETITGGGNKLTF			CASSSARDRGYEQYF		
	3	CAARFSNQFYF	Neoantigen, Melanoma	IPO9-FSS, MLL2-L8H, ZNT8_LLS, MART-1	CASSSQGISYEQYF	Neoantigen	OR14C36-FML_V6L, OR5M3-KMV, FNDC3B_L3M, GNL3L_R4C, MRM1
	4	CARTNSGNTPLVF	Neoantigen	SEC24A-P5L	CASSSGHDGPTDTQYF	Melanoma	MART-1
	5	CAVTWGQAGTALIF	Neoantigen	LCP1-NLF, FNDC3B-L3M	CASSSTSGYNEQFF		
	6	CVFAINFNKFYF	Neoantigen	HTR1F-LVM_V2M	CASSLIGAGELFF	Melanoma	IGRP_VLF
	7	CAVNAGWTGNQFYF			CSARGGISGPLNEKLFF		
	8	CAVTDDYKLSF			CASRQETGVKNEQYF		
	9	CAVNKNYDYKLSF	Neoantigen	CNKSR1-SLA_A9V, PHKA2-LLS, DCHS1-TLF_I5M			
	10	CVFAINFNKFYF	Neoantigen	HTR1F-LVM_V2M	CASSLGNEQFF		
SCC4	1	CAGGRSSNTGKLIF			CASRTGAGATEAFF		
	2	CATDGVEYGNKLVF	Neoantigen	SEC24A_P5L, SMARCD3	CASSLVSGGNEQYF		
	3	CVVSDRYNNARLMF	DM1		CSAREGTGGLSYEQYF		
	4	CAPLGSGGYQKVTF	Neoantigen	PIGN-FLT_P7H	CASSFQGGWNTEAFF	Neoantigen	APBB2-VQY_L7F
	5	CAVVRTGTASKLTF	Neoantigen	C1S-9_N1H, ST6GALNAC2-LLF_Y6H	CASSPNYSNQPQHF	Neoantigen	GNL3L_R4C
	6	CAPLGSGGYQKVTF	Neoantigen	PIGN-FLT_P7H	CASSFQGGWNTEAFF	Neoantigen	APBB2-VQY_L7F
	7	CAVRVPPWDYKLSF	Neoantigen	BAIAP3-ILN, NOS1-FID	CASSLRGVAKNIQYF		
	8	CAASTDYGQNFVF			CASSLTGLTEAFF		
	9				CASRTGAGATEAFF		

	10	CAVNSPPGNEKLTF	Melanoma, Neoantigen	MART-1, CCM2-YML_R6H, MLL2	CASSQSEGRSDTQYF		
SCC5	1	CAVKDSNYQLIW	Neoantigen, Lymphoma	OR14C36-FML_V6L, BCL9L-FVY, OR5M3-KMV, WDR46-T3I	CSGTTGGIAEAFF		
	2	CAMTPSGGSYIPTF	Neoantigen	KCNB2-LLA_P6T, ZNF827-NLF	CASSPRDGNYEQYF		
	3	CAATNYGQNFVF	Influenza, Melanoma	Matrix protein (M1), MART-	CASSVNGGEHNEQFF		
	4	CASFPRDRVANYGYTF	Neoantigen	RYR3-VLN_E6K, VN1R5_MII_S7Y, SNX24_P6L, PABPC1-R5Q	CASSLGGGFYNEQFF		
	5	CAVRGSYIPTF	Neoantigen	ZNT8-LLS	CASGSDGNTDTQYF	Melanoma, Clear Cell Renal Carcinoma, Lung Cancer, Neoantigen	mutated CDKNA2, NY-ESO-1f, INTS1-VLL_L3F
	6	CAVSLYNFNKFYF	Neoantigen	CD47-GLT_V6F, MLL2-L8H	CASSDLTTNTGELFF	Neoantigen, Melanoma	OR5M3-KMV, CD1_LLG, MART-1
	7	CALSEATTDSWGKFQF			CASSSRGLSYNEQFF		
	8	CAMTPDNYGQNFVF	Neoantigen	OR5M3-KMV, OR5M3-KMV_T8N, HAUS3-ILN_T7A	CSARAMTEETGELFF		
	9	CVVNFGDSSYKLIF			CASSFMGRRNSPLHF		
	10	CALAGGTSYGKLTF	Neoantigen, Melanoma	OR14C36-FML_V6L, PIGN-FLT_P7H, MART-1	CSGTTGGIAEAFF		
TSCC1	1	CAVNSNYQLIW	Neoantigen	PIGN-FLT_P7H	CASRATLGSGTEQYF	Clear Cell Renal Carcinoma, Neoantigen	FNDC3B
	2				CASRATLGSGTEQYF	Clear Cell Renal Carcinoma, Neoantigen	FNDC3B
	3	CAMRELNRDDKIIF			CASSFHDRADEKLFF		
	4	CAMRGRSYNTDKLIF	Neoantigen	FNDC3B-VVL_L3M, ITIH6-RLG_G3V, GOLGA3-SLD_P4L, ITIH6-RLG	CASSPQGRVTEAFF		
	5	CALSEADGANNLFF	Neoantigen	NSDHL_A9V	CASSIPSRAGGDNEQFF		
	6	CLVGGPGTYKYIF	Neoantigen	CHD8-KLN	CATSGTSTLYNEQFF		
	7	CAVKRTGNQFYF	Breast Cancer, Melanoma	GP100_IMD	CASRPTPYEQYF	Neoantigen	CHD8-KLN_P7A, MRM1, AGL_GLI
	8	CAASIGGTASKLTF	Melanoma	MART-1	CASSFLGGRLSDTQYF	Neoantigen	FNDC3B
	9	CAMLDTGNQFYF	Melanoma, Neoantigen	MART-1, PLXNB1-VLF, PTCHD4-HQL, ATP6AP1-KLG_G3W, NSDHL_A9V	CASSLDREDEKLFF		
	10	CAMRDYGQNFVF	Melanoma	OR5M3-KMV_T8N	CASSDPGGTGELFF	CMV	OR5M3-KMV_T8N
TSCC2	1	CAYKDRGSTLGRLYF	Neoantigen, Melanoma	TRPC1-MLL_Q5H, GP100-IMD	CSARDENRGWETQYF		
	2	CATEEDNRLAF			CSVDLAGGYEQYF		
	3	CASSLAAETSYEQYF			CAVNSRGSTLGRLYF		
	4	CAVRDMPPGGFKTIF	Melanoma	GP100_IMD	CASSSGLAGIYEQYF	Melanoma	MART-1
	5	CAMGNSGGSNYKLTF			CAISGGGAINEKLFF	Neoantigen	USP28-LII_C5F, GLRA1-LIF
	6				CASSFTRLYNEQFF	Neoantigen	TRIM16-RMA_R1T
	7	CAMGNSGGSNYKLTF			CAIMPGFLVGAYEQYF	Melanoma	MART-1

	8	CAVSLEEKTSYDKVIF			CAIMPGFLVGAYEQYF	Melanoma	MART-1
	9	CAVGARRGADGLTF	Melanoma	MART-1	CASSPRDRAYEQYF	Neoantigen	TRIM58-YMV_V3F, ERBB2, IV-GIL
	10	CAVGAIDTNAGKSTF			CASSTSGGFGDEQFF		
TSCC3	1	CAVNSNYQLIW	Neoantigen	PIGN-FLT_P7H	CASRATLGSGTEQYF	CMV, RA, EBV, Influenza, Clear Cell Renal Carcinoma, Allergy, HIV, Neoantigen, HCV	pp65, BZLF-1, Matrix protein (M1), Gag polyprotein RQ13, FNDC3B, HCV-KLV
	2	CAVKRTGNQFYF	Breast Cancer, Melanoma	GP100_IMD	CASRPTPYEQYF	Neoantigen	CHD8-KLN_P7A, MRM1, AGL_GLI
	3				CASRATLGSGTEQYF	Clear Cell Renal Carcinoma, Neoantigen	FNDC3B
	4	CAMRDYGQNFVF	Neoantgen	OR5M3-KMV_T8N	CASSDPGGTGELFF	Neoantigen	OR5M3-KMV_T8N
	5	CAMRGRSYNTDKLIF	Neoantigen	FNDC3B-VVL_L3M, ITIH6-RLG_G3V, GOLGA3-SLD_P4L, ITIH6-RLG	CASSPQGRVTEAFF		
	6	CLVGGPGTYKYIF	Neoantigen	CHD8-KLN	CATSGTSTLYNEQFF		
	7	CAVDDYKLSF	Neoantigen	CD1-LLG	CASRGDYQETQYF		
	8	CAVRDESSNTGKLIF	Melanoma	GP100_IMD	CASREQGGTEAFF	Neoantigen	VN1R5_MII_S7Y
	9	CAMRELNRDDKIIF			CASSFHDRADEKLFF		
	10	CAVMDSGYSTLTF	Neoantigen	FNDC3B-VVL_L3M, WDR46-FLT_T3I, ZNT8_VMI	CASSTSGTNYEQYF	Melanoma, Neoantigen	MART-1, ATP6AP1-KLG_G3W
TSCC4	1	CAVKRTGNQFYF	Breast Cancer, Melanoma	GP100_IMD	CASRPTPYEQYF	Neoantigen	CHD8-KLN_P7A, MRM1, AGL_Gli
	2	CAVNSNYQLIW	Neoantigen	PIGN-FLT_P7H	CASRATLGSGTEQYF	Clear Cell Renal Carcinoma, Neoantigen	FNDC3B
	3	CAMREPRSNDYKLSF			CASRGGTGGGKTQYF	Neoantigen, Melanoma	RYR3-VLN_E6K, AKAP13, MART-1
	4				CASRATLGSGTEQYF	Clear Cell Renal Carcinoma, Neoantigen	FNDC3B
	5	CLVGGPGTYKYIF	Neoantigen	CHD8-KLN	CATSGTSTLYNEQFF	Breast Cancer, Neoantigen, Melanoma	IVPA_FMY, MART-1
	6	CAMRELNRDDKIIF			CASSFHDRADEKLFF		
	7	CAFMKPNDYKLSF	Melanoma, Neoantigen, Lymphoma	MART-1, pp65, CNKSR1-SLA_A9V, BCL9L-FVY_T6I, PRSS16-LLL_L1Q, HCV-KLV(PE), APBB2-VQY_L7F, PHKA2-LLS, ITIH6-RLG, HCV-KLV	CASSRRLDRQGTEAFF		
	8	CALTLTGTASKLTF	Neoantigen	NSDHL-A9V	CASSPNYSNQPQHF	Neoantigen	GNL3L_R4C
	9	CAMRGRSYNTDKLIF	Neoantigen	FNDC3B-VVL_L3M, ITIH6-RLG_G3V, GOLGA3-SLD_P4L, ITIH6-RLG	CASSPQGRVTEAFF		
	10	CAVDDYKLSF	Neoantigen	CD1-LLG	CASRGDYQETQYF		
TSCC5	1	CAVSGDTGRRALTF			CASSVTNQETQYF	Melanoma, Neoantigen	Mutated CDKNA2, GANAB-S5F, NSDHL-A9V
	2	CAASWGKAAGNKLTF			CASSLVGEGNTEAFF	Neoantigen	GANAB-S5F, GNL3L_R4C
	3	CAAKGIGGSYIPTF	Neoantigen	NSDHL-A9V, WDR46_T3I	CASSPSDDKNIQYF	Melanoma	MART-1
	4	CAASSNQGGKLIF	Neoantigen	HTR1F-9_V1M, HAFP_FMN	CASSLGPNYEQYF	Neoantigen	ATP6AP1-KLG_G3W

	5	CALTPPGHSSASKIIF	Neoantigen	TRIM58-VLA, ERBB2-ALI_H8Y	CASSRLAGEGETQYF		
	6	CAFCFYNNNDMRF			CASSLRGANVLTF		
	7	CAARSEYGNKLVF	Melanoma, Neoantigen	MART-1,TRPV4-FMI_A6T, TBX3-GMG_T8M	CASTLQDGGNSPLHF	Breast Cancer	
	8	CAFNNNDMRF	Melanoma	MART-1	CSARDSGTTVYEQYF		
	9	CAAFDGDSWGKLQF	Neoantigen	ITIH6-RLG, ZNT8_LLS	CATSRGGTDTQYF		
	10	CLVGDPIVQKAAGNKLTF	Neoantigen	GNL3L_R4C	CASSPSSATNEKLFF	Neoantigen, Melanoma	RYR3-VLN, GLRA1-LIF, MART-1
TSCC6	1	CAVIARKAGTALIF			CSAKAGPGETQYF	Melanoma	MART-1
	2	CAVRFRYNDMRF	Neoantgen	PELP1-RLH_L7F, PXDNL-SIL_S1F	CASSLWTGNTEAFF	Neoantigen	FNDC3B
	3	CAVVNRDDKIIF	Neoantigen	WDR46	CAWSWMSSYNEQFF		
	4	CAMREGEIGDYKLSF			CASSPQGATEAFF	Neoantigen, Melanoma	SNPG_IML, MART-1
	5	CAASGASGGYNKLIF			CASSYSAPGYEQYF	Melanoma, Neoantigen	MART-1, HAUS3-ILN_T7A, GNL3L_R4C, IE-1
	6	CAEGDNTDKLIF	Neoantigen	ATP6AP1-KLG_G3W, GNL3L_R4C, SEC24A	CASQTGNQPQHF		
	7				CSAKAGPGETQYF	Melanoma	MART-1
	8	CAVILRGSQGNLIF	Neoantigen	NOS1-FID	CAISLDSQGGNGYTF		
	9	CASAPSGNTPLVF	Melanoma	MART-1	CASSLGTYNEQFF	Neoantigen	HTR1F-9_V1M, ALSPVIPLI
	10	CAGPITQGGSEKLVF	Tumor	NY-ESO-1	CASSRGQAYEQYF		
